# Luspatercept in Transfusion‐Dependent Thalassemia With Red Blood Cell Antibodies: A Case Series

**DOI:** 10.1002/jha2.70320

**Published:** 2026-05-27

**Authors:** Nattiya Teawtrakul

**Affiliations:** ^1^ Division of Hematology Department of Internal Medicine Srinagarind Hospital Faculty of Medicine Khon Kaen University Khon Kaen Thailand

**Keywords:** alloimmunization, autoantibody, luspatercept, thalassemia, transfusion

## Abstract

Red blood cell (RBC) antibodies complicate transfusion support in transfusion‐dependent thalassemia (TDT), and data on luspatercept in this setting remain limited. We retrospectively reviewed seven adults with TDT and RBC antibodies treated with luspatercept at Srinagarind Hospital from January 2025 to January 2026. Baseline transfusion need was six packed RBC units/12 weeks in all patients. During weeks 13–24, six patients achieved a clinically meaningful reduction, with a median decrease of three units/12 weeks (range, 2–5; median 50.0%). Responses were greatest without warm autoantibodies. No new alloantibodies or serious adverse events were observed.

**Trial Registration**: The authors have confirmed clinical trial registration is not needed for this submission.

## 1 Introduction

Chronic red blood cell (RBC) transfusion remains central to the management of transfusion‐dependent thalassemia (TDT), but repeated donor exposure increases the risk of alloimmunization and, less commonly, autoantibody formation. Alloantibodies have been reported in approximately 6%–22% of transfused patients and are frequently directed against Rh and Kell antigens. Their development is influenced by cumulative transfusion exposure, splenectomy, and donor–recipient antigen disparity [1, 2, 3]. Warm autoantibodies may coexist in heavily transfused patients, shorten RBC survival, and obscure clinically significant alloantibodies during compatibility testing [1, 4, 5]. Together, alloantibodies and autoantibodies create a challenging clinical situation: compatible units may be difficult to obtain, transfusions may be delayed, and transfused RBCs may have reduced survival.

Luspatercept is an erythroid maturation agent that improves late‐stage erythropoiesis and reduces transfusion burden in adults with TDT [6, 7]. In the BELIEVE trial and subsequent clinical guidance, luspatercept reduced RBC requirements in a clinically meaningful proportion of patients [6, 7]. A post hoc subgroup analysis of patients with HbE/β‐thalassemia reported responses broadly consistent with the overall trial population, an important observation for Southeast Asian cohorts in which HbE/β‐thalassemia predominates [8]. However, patients with complex RBC antibody profiles remain underrepresented in published data, despite being among those with the greatest transfusion‐related need. We, therefore, evaluated the efficacy, safety, and practical clinical implications of luspatercept in adults with TDT complicated by RBC antibodies, including alloantibodies and/or warm autoantibodies.

## 2 Methods

We performed a retrospective review of adults with TDT treated with luspatercept at Srinagarind Hospital, Khon Kaen University, Thailand, between January 2025 and January 2026. Eligible patients were aged 18 years or older, required regular transfusion support, and had clinically significant RBC antibodies, including alloantibodies and/or warm autoantibodies. Thalassemia diagnosis was established by hemoglobin analysis and molecular testing. Clinical data were extracted from medical records, including genotype, splenectomy status, spleen size, antibody profile, pretransfusion hemoglobin, transfusion requirement, iron chelation, serum ferritin, and adverse events. Alloantibodies and autoantibodies were identified using standard gel‐column agglutination methods.

Packed RBC transfusions were leukocyte‐depleted units prepared by the hospital blood bank. The routine baseline transfusion schedule for all patients was two packed RBC units per month, equivalent to six units per 12 weeks. During weeks 13–24, posttreatment transfusion burden was reported as whole RBC units per 12‐week interval. Percentage reduction during weeks 13–24 was calculated from the posttreatment unit count using the baseline requirement of six units per 12 weeks. The best reduction over any 12‐week interval after luspatercept initiation was reported separately as a percentage endpoint. Luspatercept was administered subcutaneously at 1.0 mg/kg every 3 weeks, with escalation to 1.25 mg/kg when clinically indicated.

Response was assessed during weeks 13–24 and over any 12‐week interval after treatment initiation. An excellent response was defined as at least a 50% reduction in transfusion requirement with a hemoglobin increase of at least 2.0 g/dL; a good response as at least a 33% reduction in transfusion requirement or a hemoglobin increase of at least 1.0 g/dL; and a satisfactory response as lesser but clinically evident improvement.

No patient received new corticosteroids, intravenous immunoglobulin, rituximab, cyclosporine, or other immunosuppressive treatment specifically for active autoimmune hemolytic anemia during the luspatercept assessment period. Folate supplementation and iron chelation were continued according to institutional practice. Individual chelation regimens and ferritin values are summarized in Table 1. Paired ferritin data were available for all seven patients; median ferritin changed from 975 ng/mL (IQR, 777–2500) at baseline to 1078 ng/mL (IQR, 503–2755) at approximately Week 24. Routine thrombophilia screening was not performed in the absence of prior thrombosis or a strong clinical indication. Adverse events were graded using the Common Terminology Criteria for Adverse Events, version 5.0. The study was approved by the Ethics Review Board for Human Research, Faculty of Medicine, Khon Kaen University (HE691085).

**TABLE 1:** Baseline clinical characteristics, transfusion outcomes, safety, and iron monitoring.

Pt	Sex/age	Genotype	Spleen	RBC antibodies	Baseline Hb	Mean Hb weeks 13–24	Baseline units/12 week	Reduction in transfusion burden, units/12 week (%)	Posttreatment transfusion burden, units/12 week	Time to response	Adverse events	Iron chelation	Ferritin baseline/week 24 (ng/mL)
P1	F/40	beta CD41/42 (‐TTCT)/beta E; alpha alpha/alpha alpha	No (spleen 13 cm)	Anti‐E	7.4	8.10	6	4 units (66.7%)	2	6 weeks	Headache Grade 1	Deferasirox 20 mg/kg/day	975/1078
P2	F/38	beta CD41/42 (‐TTCT)/beta E; alpha alpha/alpha alpha	No (spleen 12 cm)	Anti‐E	6.9	8.05	6	4 units (66.7%)	2	4 weeks	None	Deferiprone 60 mg/kg/day	777/355
P3	F/41	beta CD41/42 (‐TTCT)/beta E; alpha alpha/alpha alpha	Yes	Anti‐E, anti‐c, anti‐Le^a^, warm autoAb	7.9	8.14	6	2 units (33.3%)	4	24 weeks	Bone pain/arthralgia Grade 1–2	Deferiprone 50 mg/kg/day	438/503
P4	M/25	beta CD41/42 (‐TTCT)/beta E; alpha alpha/alpha alpha	No (spleen 8 cm)	Anti‐Di^a^, warm autoAb	6.4	7.30	6	3 units (50.0%)	3	6 weeks	None	Deferasirox 30 mg/kg/day	2166/2129
P5	F/38	beta E/beta A; –SEA/alpha CS alpha	No (spleen 7 cm)	Warm autoAb	6.6	7.13	6	3 units (50.0%)	3	12 weeks	Fatigue Grade 1	Deferasirox 30 mg/kg/day	2500/2755
P6	F/34	beta CD71/72 (+A)/beta E; alpha alpha/alpha alpha	No (spleen 3 cm)	Anti‐E, anti‐Mi^a^	6.6	8.60	6	5 units (83.3%)	1	6 weeks	None	Deferiprone 70 mg/kg/day	838/707
P7	F/37	beta IVSII‐654 (C>T)/beta E; alpha alpha/alpha alpha	No (spleen 5 cm)	Anti‐E, warm autoAb	7.8	8.40	6	3 units (50.0%)	3	6 weeks	Fatigue Grade 1	Deferiprone 90 mg/kg/day + deferasirox 30 mg/kg/day	11,482/7415

*Note*: Baseline transfusion burden was standardized as two packed RBC units per month, equivalent to six units per 12 weeks. Percentage reductions were calculated from the baseline of six units per 12 weeks. Ferritin values represent baseline and approximately Week 24; interpretation is descriptive because patients continued concurrent iron chelation.

Abbreviations: Hb, hemoglobin; RBC, red blood cell; wk, week.

## 3 Results

Seven patients were included; six were female, and the median age was 38 years (range, 25–41). Six patients had HbE/β‐thalassemia, and one had compound heterozygous HbH/HbE disease. One patient had undergone splenectomy, whereas the remaining patients had intact spleens with variable splenomegaly. The RBC antibody profile included anti‐E (*n* = 5), anti‐c (*n* = 1), anti‐Di^a^ (*n* = 1), anti‐Mi^a^ (*n* = 1), and anti‐Le^a^ (*n* = 1). Four patients had warm autoantibodies, including one patient with a warm autoantibody without a documented alloantibody. Baseline median pretransfusion hemoglobin was 6.9 g/dL.

**FIGURE 1:**
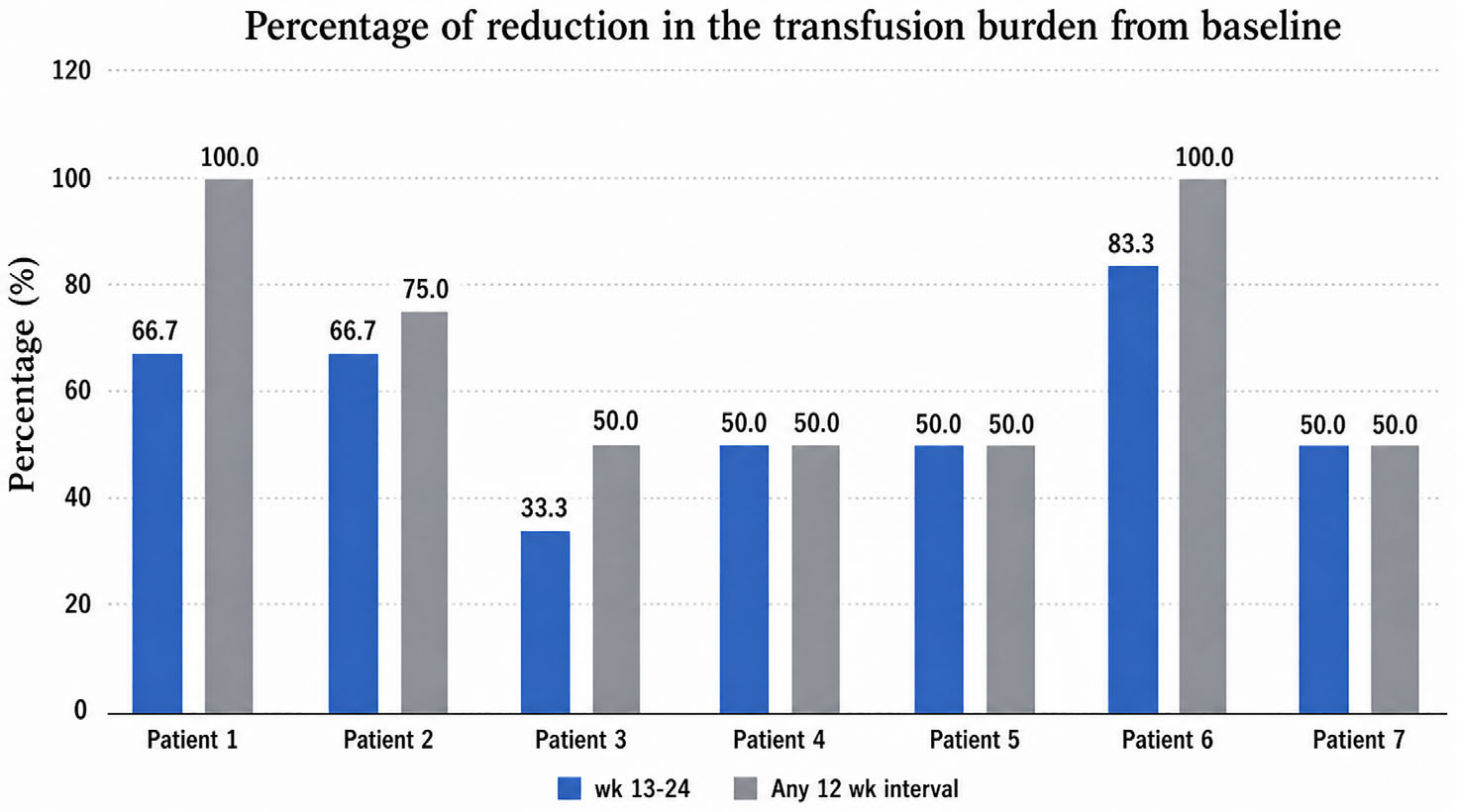
Percentage reduction in red blood cell transfusion requirement during weeks 13–24 and over any 12‐week interval after initiation of luspatercept.

Six of seven patients achieved a clinically meaningful response during weeks 13–24. Posttreatment transfusion burden decreased from six units per 12 weeks at baseline to a median of three units per 12 weeks (range, 1–4). This corresponded to a median absolute reduction of three units per 12 weeks (range, 2–5) and a median percentage reduction of 50.0% (range, 33.3%–83.3%). Patients without warm autoantibodies derived the greatest benefit during weeks 13–24, with reductions of 66.7%–83.3%, equivalent to 4–5 fewer units per 12 weeks. Over any 12‐week interval, these patients achieved reductions of 75.0%–100.0%. Patients with warm autoantibodies generally achieved reductions of 33.3%–50.0% during weeks 13–24, equivalent to 2–3 fewer units per 12 weeks. The least favorable response occurred in the only splenectomized patient, who had multiple alloantibodies and a warm autoantibody; this patient required four units per 12 weeks after treatment, representing a reduction of two units per 12 weeks (33.3%). Median pretransfusion hemoglobin increased from 6.9 g/dL at baseline to 8.10 g/dL during weeks 13–24. Time to response ranged from 4–24 weeks (Figure 1).

No new alloantibodies were detected during follow‐up. Treatment was well tolerated. Fatigue occurred in two patients, headache in one, and bone pain/arthralgia in one; all were Grade 1–2 and self‐limited. No thromboembolic events, clinically significant hypertension, thrombocytosis, injection‐site reactions, hepatic toxicity, or extramedullary hematopoietic masses were observed. No patient required treatment discontinuation because of adverse events.

## 4 Discussion

This case series suggests that luspatercept may retain clinically relevant activity in adults with TDT and RBC antibodies, a subgroup in whom transfusion is often both logistically difficult and biologically inefficient. The response pattern was clinically plausible. Patients without warm autoantibodies achieved the greatest reductions in transfusion burden when whole‐unit transfusion counts were used, whereas the least favorable response occurred in the splenectomized patient with multiple alloantibodies and a concurrent warm autoantibody. These findings suggest that ongoing immune‐mediated hemolysis and greater immunohematologic complexity may blunt the apparent benefit of improved erythroid maturation.

The predominance of HbE/β‐thalassemia in our cohort is relevant. The BELIEVE HbE/β‐thalassemia subgroup analysis reported responses consistent with the broader trial population, supporting the biological plausibility of benefit in this genotype [8]. In our series, however, genotype alone did not appear to explain response; autoantibody status, splenectomy, and antibody complexity appeared more clinically informative. These observations should be interpreted cautiously because of the small sample size, but they may help frame future studies of predictors of luspatercept response in highly transfusion‐challenged populations.

Iron metabolism also deserves attention. Reducing transfusion burden may lessen transfusional iron input, while luspatercept has been reported to stimulate erythropoiesis, increase iron utilization, and redistribute body iron [9]. In this series, ferritin decreased in four of seven patients, including the patient with the highest baseline ferritin level; however, median ferritin changed only modestly from 975 ng/mL at baseline to 1078 ng/mL at approximately Week 24. These trends should be interpreted descriptively because the follow‐up was short, and all patients continued chelation therapy. Ferritin is also an imperfect short‐term marker and may be influenced by inflammation, chelation adherence, and baseline iron burden. Longitudinal liver iron concentration or cardiac T2* assessment would be preferable in larger prospective studies.

The absence of serious toxicity is reassuring but should not be overinterpreted. Real‐world data and recent commentaries emphasize that the benefit of luspatercept is clinically meaningful, but careful monitoring remains necessary [10, 11, 12]. Particular attention should be given to thrombosis, hypertension, thrombocytosis, and possible expansion of extramedullary hematopoietic masses, especially in splenectomized patients or those with preexisting masses [11, 12, 13]. In our practice, clinical monitoring was performed at each visit, and imaging was guided by symptoms rather than performed routinely.

From a transfusion‐service perspective, reducing donor exposure is a practical advantage in patients with RBC antibodies. Luspatercept does not replace extended antigen matching, careful antibody investigation, or immunosuppressive therapy when active autoimmune hemolysis is present. Its role is best considered adjunctive: by reducing transfusion requirement and improving pretransfusion hemoglobin, it may reduce future antigen exposure and improve the feasibility of transfusion planning. Exact days of transfusion delay and use of rare donor or frozen blood resources were not systematically captured in this retrospective review.

This study is limited by its retrospective design, small sample size, short follow‐up, and incomplete iron‐overload assessment. Nevertheless, it addresses a clinically important subgroup for whom prospective data are scarce. Luspatercept appears to be a useful adjunct in selected adults with TDT and RBC antibodies, particularly those without active autoantibody‐mediated hemolysis. Larger prospective studies should define the durability of response, effects on new antibody formation, optimal safety monitoring, and integration with transfusion‐service strategies for highly immunized patients.

## Funding

The author has nothing to report.

## Ethics Statement

This study was approved by the Ethics Review Board of Human Research, Faculty of Medicine, Khon Kaen University, Khon Kaen, Thailand (Approval Code: HE691085).

## Conflicts of Interest

The author declares no conflicts of interest.

## Data Availability

The data that support the findings of this study are available from the corresponding author upon reasonable request, subject to institutional and ethical restrictions.
